# Draft Genome Sequence of the Phenol-Degrading Bacterium *Pseudomonas putida* H

**DOI:** 10.1128/genomeA.00936-15

**Published:** 2015-08-20

**Authors:** Paula Vizoso, Nicolas Pacheco, Macarena Bastias-Molina, Claudio Meneses, Ignacio Poblete-Castro

**Affiliations:** a Universidad Andrés Bello, Facultad de Ciencias Biológicas, Centro de Biotecnología Vegetal, Santiago, Chile; b FONDAP Center for Genome Regulation, Santiago, Chile; c Universidad Andrés Bello, Biosystems Engineering Laboratory, Center for Bioinformatics and Integrative Biology, Facultad de Ciencias Biológicas, Santiago, Chile

## Abstract

In this study, we report the draft genome of *Pseudomonas putida* H, a well-known bacterium capable of degrading various aromatic compounds. Its genome size is 6,065 Mbp with a GC content of 61.6%. This work will aid future studies on this versatile bacterium.

## GENOME ANNOUNCEMENT

Members of the genus *Pseudomonas* are capable of colonizing a broad range of environments such as soil, water, plants, and animal tissues (1). This feature is due to their genetic plasticity and high metabolic versatility to cope with the changing nutrient availability in nature (2). Beyond their first use as biological agents to treat contaminated environments, *Pseudomonas putida* strains are important cell factories for the production of value-added chemicals (3). One of the biggest challenges in industrial biotechnology is the use of toxic compounds to synthesize chemicals that can be directly used for obtaining bioproducts (4). Phenolic compounds are produced in numerous industrial processes such as in the petrochemical, pharmaceutical, textile, and steel industries, where their removal from wastewater streams is currently a major environmental concern (5). In this study, we report the draft genome sequence of *Pseudomonas putida* H, a well-known phenol-degrading bacterium (6). 

The DNA library construction and sequencing using the Illumina MiSeq platform (Plant Biotechnology Center, Universidad Andrés Bello). We designed a 540-bp paired-end library to generate paired-end sequencing reads of 2 × 300 bp. A total of 12,356,854 clean reads (totaling 12.3 Mb) were generated. The sequence reads were trimmed based on their quality scores, (Q ≥ 30). The *de novo* assembly was performed using CLC Genomics Workbench version 6.5.2 (length fraction, 0.5; similarity fraction, 0.9) and SOAPdenovo version 1.05, which yielded 55 contigs (>2,000 bp). The largest contig was 549,561 bp in length. *Pseudomonas putida* H has a total genome size of 6,065,319 bp. The G+C content of the assembly is 61.6%, and the *N*_50_ is 212,615. Annotation revealed 5,653 coding sequences (CDSs), including 4 rRNA genes and 67 tRNA genes. Analysis of the genome of *P. putida* H shows open reading frames codifying for enzymes responsible for the catabolism of benzoate, *p*-coumarate, phenylalanine, and phenylacetate. As previously described, we did not find genes for phenol degradation in the circular chromosome of *P. putida* H; instead, they are localized in the pPGH1 plasmid (7). This work will spur our knowledge in the metabolic capabilities of *Pseudomonas putida* strains for both the remediation of aromatic compounds and the use of toxic waste materials as carbon substrates for the biotechnological production of high-value chemicals.

### Nucleotide sequence accession numbers.

This whole-genome shotgun project has been deposited at DDBJ/EMBL/GenBank under the accession number LFYQ00000000. The version described in this paper is the first version, LFYQ01000000.
